# Four human *Plasmodium* species quantification using droplet digital PCR

**DOI:** 10.1371/journal.pone.0175771

**Published:** 2017-04-19

**Authors:** Suttipat Srisutham, Naowarat Saralamba, Benoit Malleret, Laurent Rénia, Arjen M. Dondorp, Mallika Imwong

**Affiliations:** 1 Department of Molecular Tropical Medicine and Genetics, Faculty of Tropical Medicine, Mahidol University, Bangkok, Thailand; 2 Mahidol-Oxford Tropical Medicine Research Unit, Faculty of Tropical Medicine, Mahidol University, Bangkok, Thailand; 3 Laboratory of Pathogen Immunobiology, Singapore Immunology Network (SIgN), Agency for Science Technology and Research (A*STAR), Biopolis, Singapore, Singapore; 4 Department of Microbiology and Immunology, Yong Loo Lin School of Medicine, National University of Singapore, National University Health System, Singapore, Singapore; 5 Centre for Tropical Medicine and Global Health, Churchill Hospital, University of Oxford, Oxford, United Kingdom; Centro de Pesquisas Rene Rachou, BRAZIL

## Abstract

Droplet digital polymerase chain reaction (ddPCR) is a partial PCR based on water-oil emulsion droplet technology. It is a highly sensitive method for detecting and delineating minor alleles from complex backgrounds and provides absolute quantification of DNA targets. The ddPCR technology has been applied for detection of many pathogens. Here the sensitive assay utilizing ddPCR for detection and quantification of *Plasmodium* species was investigated. The assay was developed for two levels of detection, genus specific for all *Plasmodium* species and for specific *Plasmodium* species detection. The ddPCR assay was developed based on primers and probes specific to the *Plasmodium* genus *18S rRNA* gene. Using ddPCR for ultra-sensitive *P*. *falciparum* assessment, the lower level of detection from concentrated DNA obtained from a high volume (1 mL) blood sample was 11 parasites/mL. For species identification, in particular for samples with mixed infections, a duplex reaction was developed for detection and quantification *P*. *falciparum*/ *P*. *vivax* and *P*. *malariae*/ *P*. *ovale*. Amplification of each *Plasmodium* species in the duplex reaction showed equal sensitivity to singleplex single species detection. The duplex ddPCR assay had higher sensitivity to identify minor species in 32 subpatent parasitaemia samples from Cambodia, and performed better than real-time PCR. The ddPCR assay shows high sensitivity to assess very low parasitaemia of all human *Plasmodium* species. This provides a useful research tool for studying the role of the asymptomatic parasite reservoir for transmission in regions aiming for malaria elimination.

## Introduction

Droplet digital polymerase chain reaction (ddPCR) is a method for nucleic acid detection and quantification without using standard curves, based on water-oil emulsion droplet technology [1]. A typical ddPCR reaction consists of the ddPCR reagent, DNA sample, primers and a fluorescent probe. Using in droplet generator, all components are divided into 20,000 droplets on which the individual PCR reactions take place. Each droplet may contain one, more than one or no copies of the DNA target [2–4]. After 40 cycles of the standard PCR reaction, DNA targets in each droplet are amplified and then analysed by a droplet reader. Positive droplets which contains DNA target will show higher amplitude of the fluorescent signal than droplets which no DNA target. The DNA target concentration is calculated from the number of positive and negative droplets using Poisson statistics [2]. A number of studies on ddPCR show high sensitivity of the method and high precision for absolute quantification of DNA targets, for example in cancer [5–8], hepatitis B virus [9, 10], cytomegalovirus[11, 12], *Chlamydia trachomatis* [13, 14], *Babesia microti*, and *Babesia duncani* [15]. Comparison of ddPCR assays with real-time PCR assays showed that ddPCR assays had a higher sensitivity than real-time PCR assays [16, 17], but this was not confirmed in some other studies [11, 18]. Real-time PCR assays are widely used for detection and relative quantification of *Plasmodium* parasites.

Sensitive molecular assays for *Plasmodium* species detection include nested PCR and real-time PCR assays. These assays have a lower level of detection around 100–1,000 parasites/mL depending on the blood volume used [19, 20]. The most widely used molecular assay is quantitative real-time PCR (qPCR) assay, which has been used for species identification and relative quantification. The sensitivity of real-time PCR (qPCR) assays for detection depends on target genes and type of sample collection. Target genes include *18S rRNA* [21–27], *tRNA* [28], *cytochrome b* [29, 30], *ama1* [31] and *Stevor* [32]. Among these, *18S rRNA* gene is the most frequently used because the gene has 5 to 7 copies per *Plasmodium* genome, which improves sensitivity of detection [32]. For example, real-time PCR assay targeting *Plasmodium 18S rRNA* has a lower limit of detection of 51 parasites/mL [27]. Concentrating parasite DNA from a high blood volume for real-time PCR assays, can improve the lower limit of detection to 22 parasites/mL, a technique here named ultrasensitive polymerase chain reaction (uPCR) [33]. This uPCR assay was used in cross-sectional survey studies to showed high prevalence of asymptomatic *Plasmodium* infections in low transmission settings in SE Asia. However, in samples with very low parasitaemias, it was not possible to determine the *Plasmodium* species [34–36]. Species identification for these samples might be useful for more detailed understanding of epidemiology of each of the species. Sensitive quantification of target DNA based approach without standard curves using ddPCR might be a useful technique for this. Recently, ddPCR assay has been developed for detection and quantification of *P*. *falciparum* and *P*. *vivax* [37]. However, the detection and quantification of *P*. *malariae* and *P*. *ovale* has not been developed so far.

In this study, we developed a sensitive assay using ddPCR for detection and quantification of *18S rRNA* of *Plasmodium* species. The assay was developed for two levels of detection; *Plasmodium* genus and *Plasmodium spp*., for specifying the *Plasmodium* species. A quantitative method with increased sensitivity for *Plasmodium* genus was developed using high blood volume samples. For assessing specific *Plasmodium spp*. a high sensitive method was developed using a duplex *P*. *falciparum*/ *P*. *vivax* and *P*. *malariae*/ *P*. *ovale* ddPCR assay, in order to increase throughput compared to a singleplex method. Associated costs of the ddPCR assay were also assessed.

## Materials and methods

### Sources of DNA samples

#### *P*. *falciparum* from Fluorescence-activated cell sorting (FACS)

To develop and validate the ddPCR assay for *Plasmodium* genus detection and quantification, we used ring stage *P*. *falciparum* 3D7 cultured parasites which were synchronized and then sorted by flow cytometry to obtain precisely defined parasitaemias, as described earlier by Malleret *et al*. (2011) [38]. For this, a sample of 2,000 parasites/mL was prepared from a total of 10,000 ring stage infected cells selected by flow cytometry diluted in 5,000 mL of whole blood from a healthy volunteer from a non-endemic area and without a history of malaria infection.

#### DNA samples

Samples were collected from malaria patients presented at Buntharik district hospital, Ubon Ratchathani, of the northeastern part of Thailand, in 2015 (N = 102) and asymptomatic individuals in Pailin, Cambodia, in 2013 (N = 32). All participants in the study were adults older than 18 years; patients and asymptomatic individuals provided written informed consent before participating in the study. Ethical approvals for the study were obtained from the ethical review board of the Faculty of Tropical Medicine, Mahidol University (MUTM 2012-045-05 and MUTM 2015-032-02). In Ubon Ratchathani, symptomatic patients with *Plasmodium* infection as diagnosed by peripheral blood microscopy were included in the study. In Pailin, Cambodia, blood samples from asymptomatic villagers were obtained and tested for malaria by uPCR [33], and 32 samples with positive results by uPCR but with unidentified *Plasmodium* species were included in this study. The collection and handling of venous blood samples were performed in accordance with the Appropriate Technology in Health (PATH) guidelines [39].

We used genomic DNA samples of *P*. *falciparum*, *P*. *vivax*, *P*. *malariae* and *P*. *ovale* in which parasitaemias were known to develop and validate the duplex ddPCR assay of *P*. *falciparum/P*. *vivax* and *P*. *malariae/P*. *ovale*. Parasitaemia (in parasites/mL) was calculated as (parasites/1000 RBCs) x 30 x 125.6 x 1,000, assuming a 30% hematocrit [40]. *Plasmodium* species was confirmed by real-time PCR assay [25, 41, 42]. Artificial mixing of DNA samples for validation of ddPCR were prepared from these 4 *Plasmodium* species DNA samples.

### Preparation of DNA samples

To increase the lower limit of detection we used DNA samples from a high volume (1 mL) blood sample for use in the ddPCR assay of *Plasmodium* genus detection and quantification. For DNA extraction from the 1,000 μl of whole blood a previously described and validated method using “speed vac” was used [33]. In short, DNA samples from 1,000 μl of whole blood was centrifuged at 2,500 rpm for 10 minutes, after which plasma and buffy coat were removed to obtain packed red blood cells. DNA was extracted using a QIAamp blood minikit, after which the DNA sample was dried and concentrated in a centrifugal vacuum concentrator. After this the DNA was resuspended in 40 μl of PCR-grade water, providing a 25-timesX concentrated DNA sample where 1 μl from concentrated DNA sample corresponds to 25 μl of whole blood [33].

For the ddPCR assay for *Plasmodium* species detection and quantification, DNA samples were prepared using 200 μl of whole blood for DNA extraction with QIAamp blood minikit.

### Development and validation of the Droplet digital PCR (ddPCR) assays

**Fig 1:**
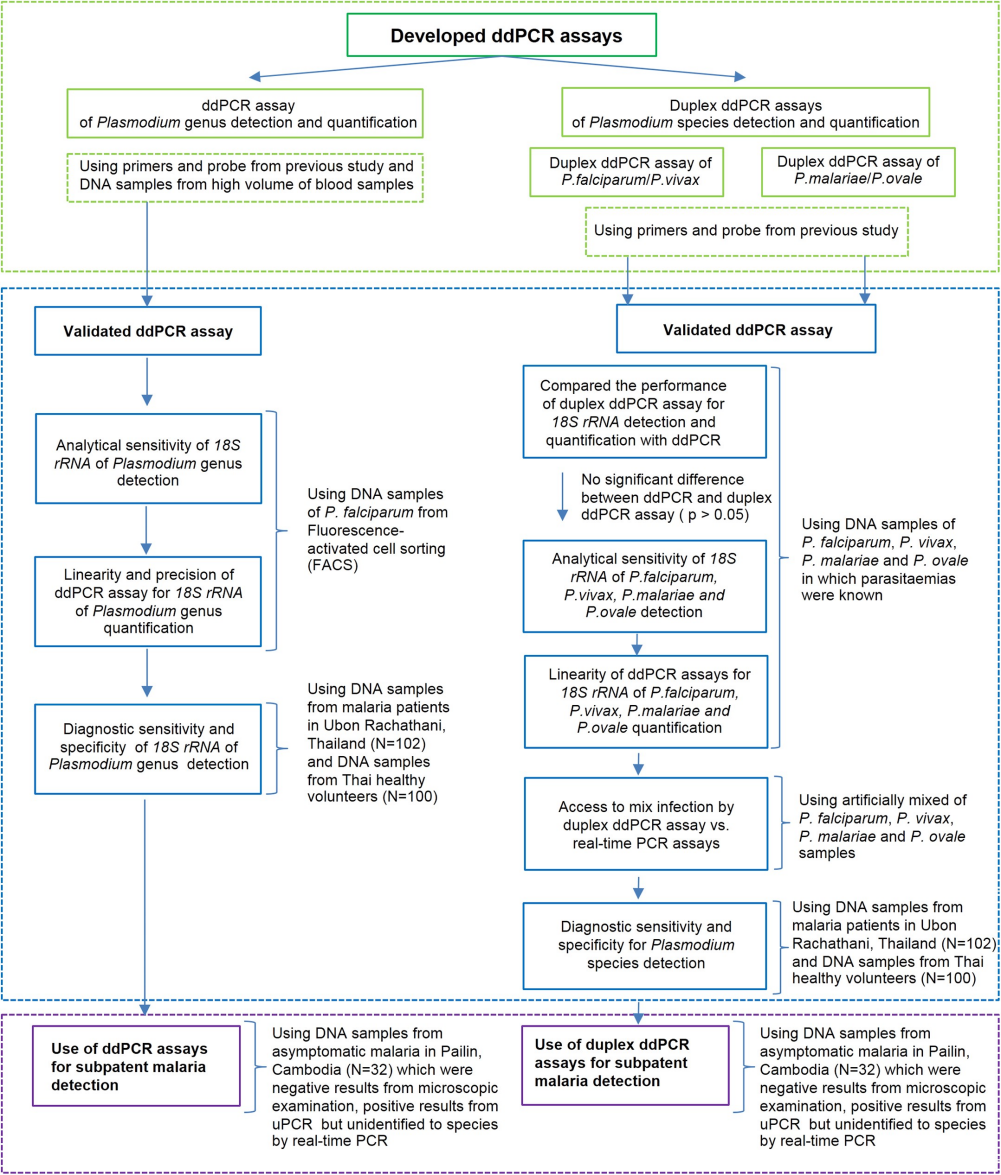
Workflow of the study.

This study aimed to develop and validate ddPCR assays for detection and quantification of *18S rRNA Plasmodium* at genus and species levels (Fig 1) following the Standards for the Reporting of Diagnostic Accuracy (STARD) [43].

#### Droplet digital PCR assay for *Plasmodium* genus detection and quantification

**Fig 2:**
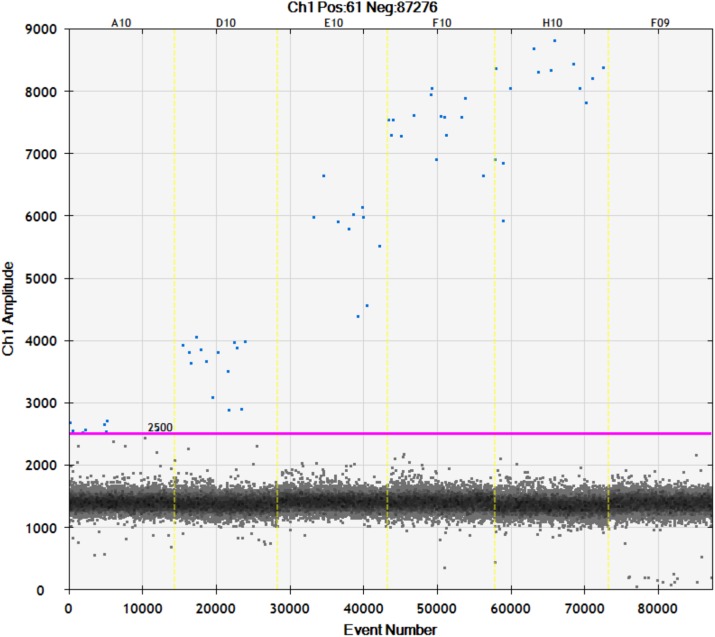
The one-dimensional (1D) ddPCR results from Bio-Rad QX100TM Droplet Reader of *18S rRNA Plasmodium* genus detection and quantification assay. The different amplitudes of positive droplets were observed when different PCR annealing temperatures were applied (A10: 65°C, D10: 62°C, E10: 60°C, F10: 59°C, H10: 57°C, F09: 60°C for negative control). Threshold for positive detection is 2500.

In this assay, the ddPCR reaction was prepared in total 20 μl per reaction containing the ddPCR reagents (10 μl of Bio-Rad 1X ddPCR Supper Mix, 0.9 μM of forward and reverse primers, and 0.25 μM of probe) and 4 μl of the DNA sample. The ddPCR reagents consist of Bio-Rad 1X ddPCR Supper Mix, forward and reverse primers, and probe. We used primers and probes previously designed to specifically target *18S rRNA* genes of *Plasmodium* genus (S1 Table). The ddPCR reactions were loaded to a Bio-Rad QX200TM Droplet generator for 12,000–20,000 droplets generation. Droplets were transferred to a PCR plate and standard PCR was performed using a Bio-Rad Thermal Cycler. To optimize the PCR annealing temperature, a temperature gradient PCR of 65°C, 62°C, 60°C, 59°C and 57°C was used. An optimized PCR annealing temperature of the assay was 60°C, which provided a clear separation between DNA positive and negative droplets (Fig 2). The conventional PCR was run with 95°C for 10 minutes, 94°C for 30 seconds and 60°C for 30 seconds in 40 cycles, and 98°C for 10 minutes. After 40 cycles of PCR, DNA targets in each droplet were amplified and then analyzed by the Bio-Rad QX200TM Droplet Reader. This provided the number of positive and negative droplets, as well as quantification of *18S rRNA* genes of *Plasmodium* genus, expressed as copies/μl of ddPCR reaction. At least two positive droplets were required for a positive test result of the ddPCR assay[37]. We converted the results of *18S rRNA* concentration from ddPCR to *18S rRNA* copies/mL of blood sample by using the formula: [*18S rRNA* copies/mL of blood = (copies/μl from ddPCR x 20 (total volume of ddPCR in μl) x 1,000)/ 4 (Total loaded DNA in μl) x 25 (25X concentrated DNA)].

The ddPCR assay was validated for analytical sensitivity, precision and linearity. To assess the analytical sensitivity (lower limit of detection) 7 standard concentrated DNA samples were used of 2,000, 400, 80, 16, 8, 4, and 2 parasites/mL of blood sorted by flow cytometry [38]. For each standard sample the ddPCR assays were performed in 16 replicates. The analytical sensitivity was determined by Probit analysis using SPSS 21.0. The precision or reproducibility of the ddPCR assay was assessed by repeated analysis of 16 replicates from samples containing 2,000, 400, 80 and 16 parasites/mL of blood, respectively, from which the relative standard deviation (%RSD) was estimated. Linearity between *18S rRNA* concentrations assessed by the ddPCR assay, repeated 16 times, and the number of parasites sorted by flow cytometry was assessed from spiked samples with 2,000, 400, 80 and 16 parasites/mL blood, respectively.

#### Droplet digital PCR assay for *Plasmodium* species detection and quantification

A duplex ddPCR assays for detection and quantification of 4 human *Plasmodium* species was developed. A duplex assay was considered cheaper and allowing higher throughput compared to a single species singleplex method. A duplex ddPCR assay targeting *18S rRNA* genes of *P*. *falciparum* and *P*. *vivax* was developed as well as an assay for *P*. *malariae* and *P*. *ovale*. The duplex ddPCR reaction was prepared in a total volume of 20 μl per reaction which contained ddPCR reagents and 4 μl of DNA sample. The duplex ddPCR reagents consisted of 10 μl Bio-Rad 1X ddPCR Supper Mix, and 2 sets of primers and probes (0.9 μM of primers and 0.25 μM of probes). Probes and primers have been described previously and have shown high sensitivity and specificity for *Plasmodium* species detection in real-time PCR assays (S1 Table). In the duplex ddPCR for species detection, primers and probes of *P*. *falciparum* and *P*. *vivax* were mixed in the same ddPCR reaction [25], and similarly for the primers and probes for *P*. *malariae* [41] and *P*. *ovale* [42] detection. The ddPCR reactions were loaded to the Bio-Rad QX200TM Droplet generator and the droplets were then transferred to the PCR plate for standard PCR using a Bio-Rad Thermal Cycler with optimized annealing temperatures. After 40 cycles of PCR, amplified DNA targets in each droplet were analyzed by the Bio-Rad QX200TM Droplet Reader. We converted the results of *18S rRNA* concentration from ddPCR to *18S rRNA* copies/mL of blood sample by using a formula: *18S rRNA* copies/mL of blood = (copies/μl from ddPCR x 20 (total volume of ddPCR in μl) x 1,000)/ 4 (Total loaded DNA in μl) x 1 (1X concentrated DNA).

The *18S rRNA* concentration (copies/mL) results from the duplex ddPCR assays were compared with results using single species reactions, using t-test statistical analysis and a 0.05 significance level.

The analytical sensitivity of the duplex method to detect *Plasmodium* species and linearity was assessed. An up to five-fold serial dilution of microscopically quantified samples from single species infected patients were used to assess the analytical sensitivity (lower limit of detection). For this DNA obtained from the serial diluted samples with *P*. *falciparum*, *P*. *vivax*, *P*. *malariae* or *P*. *ovale* was assessed by the duplex ddPCR assays in 8 replicates. The analytical sensitivity of the duplex ddPCR assays were determined by Probit analysis using SPSS 21.0. Linearity of the correlation between *18S rRNA* concentration obtained from the duplex ddPCR assay and the microscopically determined parasitaemia was assessed from 4 *P*. *falciparum*, *P*. *vivax*, *P*. *malariae* and *P*. *ovale* samples.

### Real-time PCR assays for *18S rRNA Plasmodium* species detection

To compare ddPCR *Plasmodium* species detection with real-time PCR assay as the reference method, real-time PCR assays were performed as described previously [25, 41, 42]. A total of 10 μl per reaction contained 400 nM of forward and reverse primer and 200 nM of probe, using a 60°C annealing temperature for *P*. *falciparum*, *P*. *vivax* detection and a 52°C annealing temperature for *P*. *malariae* and *P*. *ovale*. We interpreted the result as positive when the Ct-value of real-time PCR was lower than 40.

### Statistical analysis

We used SPSS 21.0 for statistical analysis and GraphPad Prism 6 for graph construction.

## Results

### *Plasmodium* genus detection and quantification

**Fig 3:**
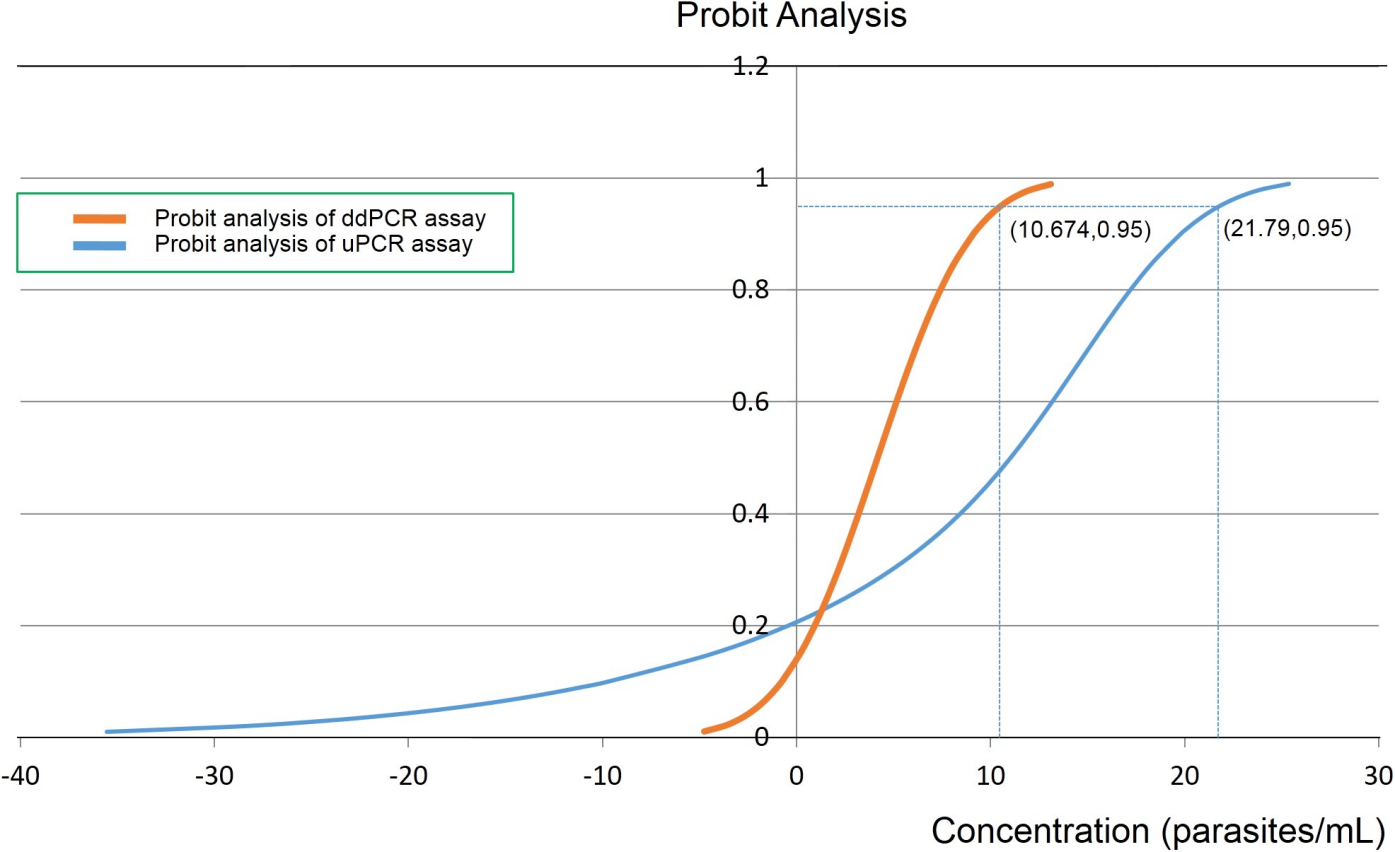
Probit analysis. The 95% probability of detecting parasitaemia as low as 10.674 parasites/mL when high volume of blood samples was applied with *Plasmodium* genus ddPCR assay.

**Fig 4:**
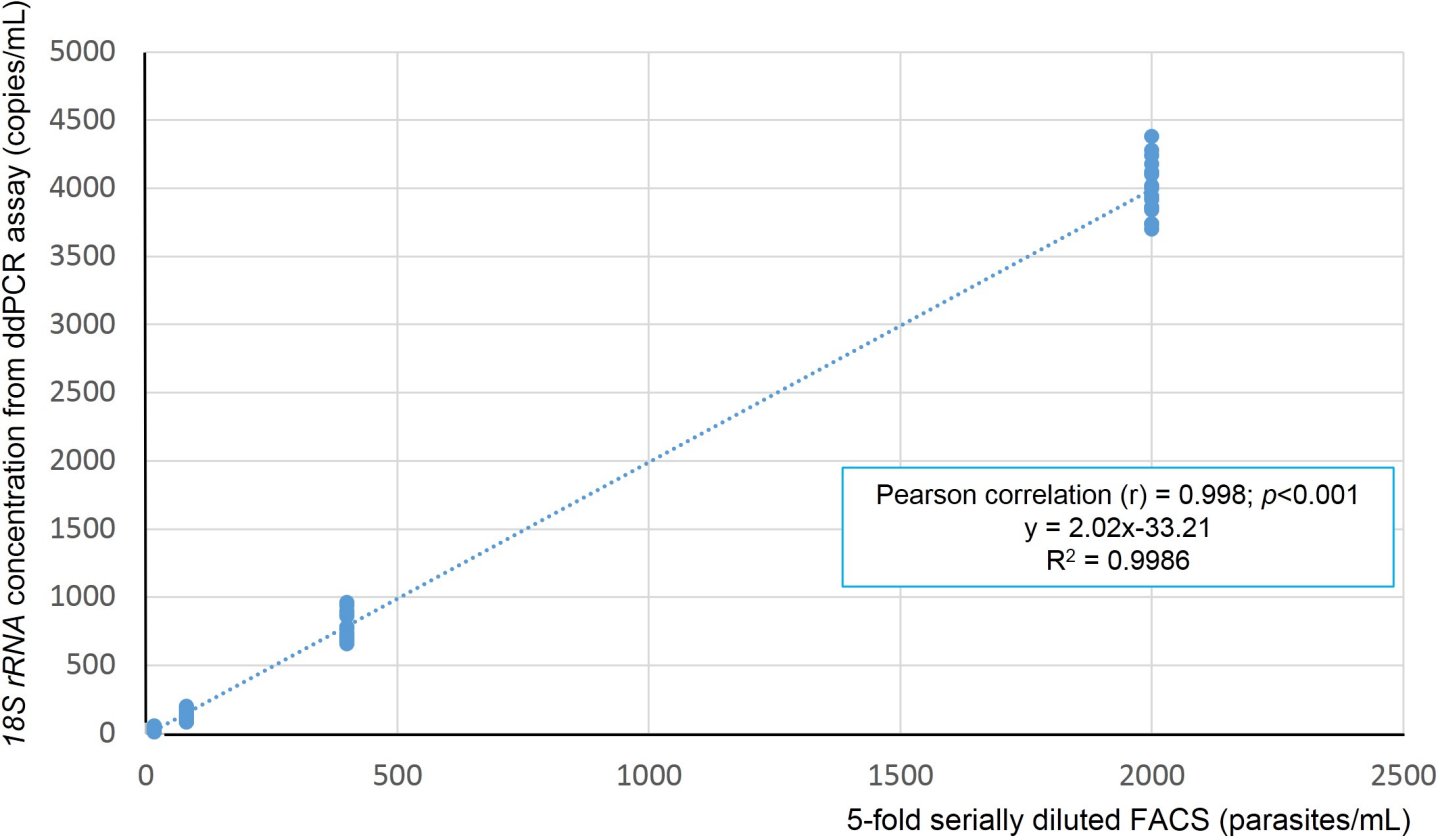
The linearity of ddPCR assays for *18S rRNA Plasmodium* genus detection and quantification. Linearity of ddPCR assay is shown using 5-fold serially diluted FACS concentrated DNA (from 2,000, 400, 80, 16 parasites/mL, respectively) and *18S rRNA* concentration from ddPCR assay (copies/mL); R^2^ = 0.9986.

The ddPCR high volume assay designed for sensitive detection had an optimized PCR annealing temperature of assay was 60°C, which provided a clearly separation between DNA positive and negative droplets (Fig 2). The ddPCR assay could detect *Plasmodium* genus in all 16 replicates containing 2,000, 400, 80 and 16 parasites/mL respectively. With very low parasitaemia samples containing 8, 4 and 2 parasites/mL, the ddPCR assay could detect parasite DNA in respectively 15, 8 and 4 times out of 16 replicates. Using Probit analysis, the threshold for 95% probability of detection was as low as 11 parasites/mL (95%, CI 8 to 19) (Fig 3). To confirm the amplification target, independent conventional PCR amplifications were used with the same set of ddPCR primers and then the PCR products were DNA sequenced. The sequencing results (N = 20) confirmed *Plasmodium 18S rRNA* as target and did not reveal any polymorphisms. Precision or repeatability of the ddPCR assay, expressed as %CV in 16 replicates is shown in S2 Table. Precision of the ddPCR assay was higher in samples with high concentrations of DNA target. The quantitative results of the ddPCR assay (as copies/μl in the ddPCR) was highly correlated with the FACS quantification (as parasites/μl) from the dilution steps: R^2^ = 0.9986 (Fig 4). The ratio between the 2 measures was around 2 for all FACS derived parasitaemias (S2 Table).

#### Duplex ddPCR for *Plasmodium* species detection and quantification

**Fig 5:**
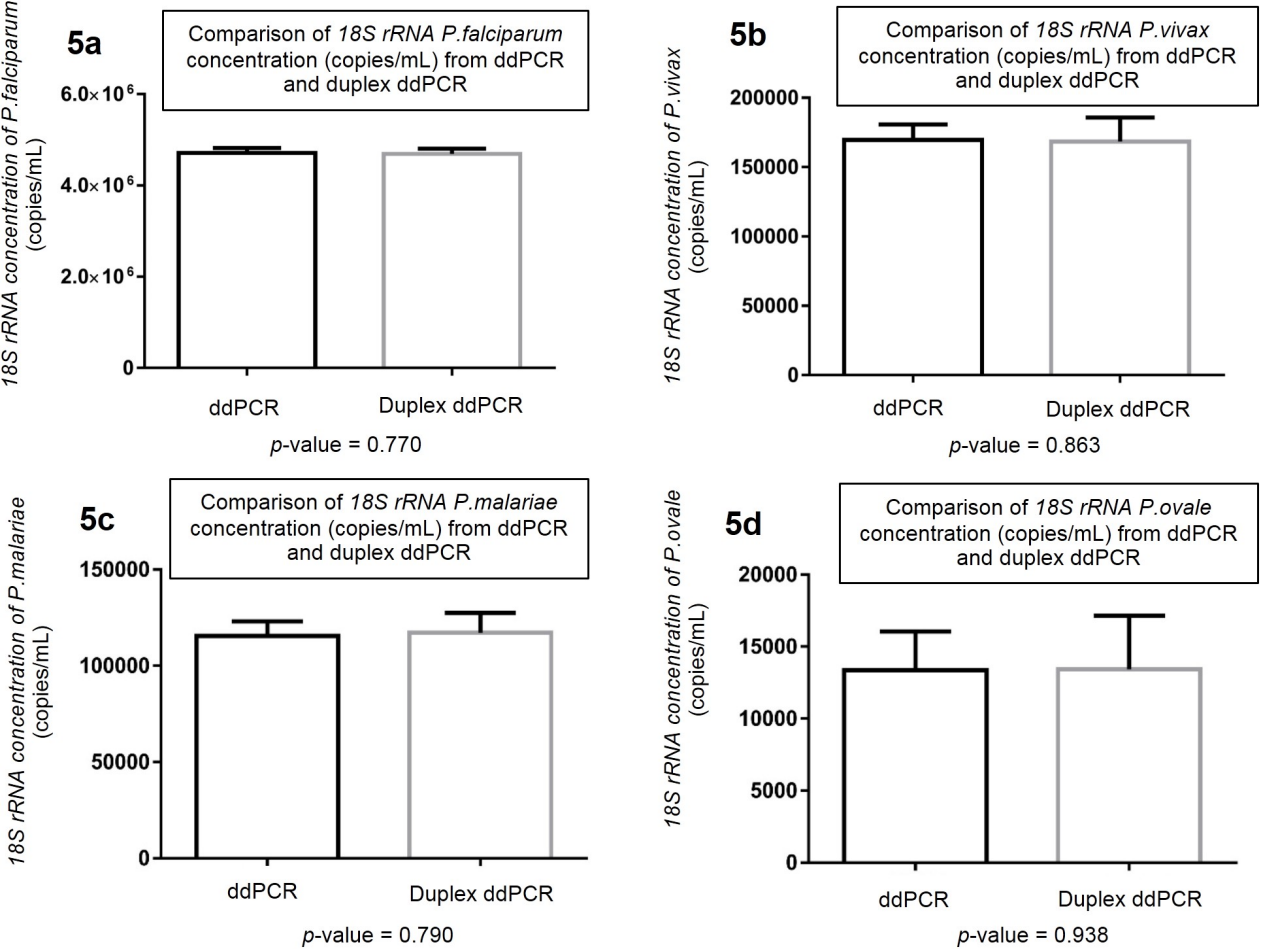
The column bar graphs show *18S rRNA* copies/mL of blood obtained from single reaction ddPCR and duplex ddPCR assays. Mean 95% CI of *18S rRNA* of *P*. *falciparum* (5a), *P*. *vivax* (5b), *P*. *malariae* (5c) and *P*. *ovale* (5d).

The duplex ddPCR for *P*. *falciparum/ P*. *vivax* and *P*. *malariae/ P*. *ovale* species detection had an optimized PCR annealing temperature of 60°C for *P*. *falciparum/ P*. *vivax* and 52°C for *P*. *malariae/ P*. *ovale* (S1 Fig). We compared the performance of duplex ddPCR assay, a reaction with 2 sets of primers and probes, to the singleplex ddPCR assay as reference. We performed this in two experiment, using mixed DNA samples of *P*. *falciparum*/ *P*. *vivax* and *P*. *malariae*/ *P*. *ovale* respectively. There was no difference in *18S rRNA* quantification between the 2 method (t-test; p = 0.770, p = 0.863, p = 0.790 and p = 0.938 for *P*. *falciparum*, *P*. *vivax*, *P*. *malariae* and *P*. *ovale* detection respectively; Fig 5). The analytical sensitivity of the duplex *P*. *falciparum/ P*. *vivax* and *P*. *malariae/ P*. *ovale* ddPCR assays estimated by Probit analysis showed a threshold for a 95% probability for detection of 162 parasites/mL (95%, CI 115 to 423) for *P*. *falciparum*, 104 parasites/mL (95%, CI 76.945 to 316.934) for *P*. *vivax*, 842 parasites/mL (95%, CI 575 to 2,431) for *P*. *malariae* and 2,825 parasites/mL (95%, CI 2,077 to 5,985) for *P*. *ovale*. Correlating the *18S rRNA* concentration (copies/μl) from duplex ddPCR assays and the number of parasites/mL in serial diluted samples showed Pearson correlation (r) = 0.998 (*p*<0.001) and R^2^ = 0.9952 for *P*. *falciparum*, r = 0.996 (*p*<0.001) and R^2^ = 0.9913 for *P*. *vivax*, r = 0.993 (*p*<0.001) and R^2^ = 0.9851 for *P*. *malariae*, and r = 0.967 (*p*<0.001) and R^2^ = 0.9357 for *P*. *ovale*.

### Comparison of duplex ddPCR assay and real-time PCR assays for mixed infections

The ability to detect minor parasite populations in artificially mixed populations of *P*. *falciparum*, *P*. *vivax*, *P*. *malariae* and *P*. *ovale* various parasite concentrations were compared between ddPCR and real-time PCR as comparator (Table 1). Both methods showed good performance for detecting minor populations of *P*. *falciparum*, *P*. *vivax* and *P*. *ovale* against a high background of *P*. *falciparum* or *P*. *vivax* DNA. However, in samples with *P*. *malariae* as a minor population against a background of high parasitaemias of *P*. *falciparum* or *P*. *vivax*, real-time PCR was unable to detect *P*. *malariae*, whereas duplex ddPCR assays could (samples No. 7–9 Table 1).

**Table 1:** Identification of mixed infection by duplex ddPCR assay in comparison to real-time (performed in quadruplicate).

Sample No.	Artificial Mixed of	Estimated parasites concentration (parasites/mL)	Real time PCR results				Duplex ddPCR results			
			*P*. *falciparum*	*P*. *vivax*	*P*. *malariae*	*P*. *ovale*	*P*. *falciparum*	*P*. *vivax*	*P*. *malariae*	*P*. *ovale*
**1.**	PF,PV	96,460.8: 1205.76	+/+/+/+	+/+/+/+	-/-/-/-	-/-/-/-	+/+/+/+	+/+/+/+	-/-/-/-	-/-/-/-
**2.**	PF,PV	19,292.16: 1205.76	+/+/+/+	+/+/+/+	-/-/-/-	-/-/-/-	+/+/+/+	+/+/+/+	-/-/-/-	-/-/-/-
**3.**	PF,PV	3,858.43: 1,205.75	+/+/+/+	+/+/+/+	-/-/-/-	-/-/-/-	+/+/+/+	+/+/+/+	-/-/-/-	-/-/-/-
**4.**	PF,PV	4,823.04: 120,576	+/+/+/+	+/+/+/+	-/-/-/-	-/-/-/-	+/+/+/+	+/+/+/+	-/-/-/-	-/-/-/-
**5.**	PF,PV	4,823.04: 24,115.2	+/+/+/+	+/+/+/+	-/-/-/-	-/-/-/-	+/+/+/+	+/+/+/+	-/-/-/-	-/-/-/-
**6.**	PF,PV	4,823.04: 4,823.04	+/+/+/+	+/+/+/+	-/-/-/-	-/-/-/-	+/+/+/+	+/+/+/+	-/-/-/-	-/-/-/-
7.*	PF,PM	482,304:128,000	+/+/+/+	-/-/-/-	-/-/-/-	-/-/-/-	+/+/+/+	-/-/-/-	+/+/+/+	-/-/-/-
8.*	PF,PM	96,460.8:128,000	+/+/+/+	-/-/-/-	-/-/-/-	-/-/-/-	+/+/+/+	-/-/-/-	+/+/+/+	-/-/-/-
9.*	PV,PM	120,576:128,000	-/-/-/-	+/+/+/+	-/-/-/-	-/-/-/-	-/-/-/-	+/+/+/+	+/+/+/+	-/-/-/-
**10.**	PV,PM	4,823.04:128,000	-/-/-/-	+/+/+/+	+/+/+/+	-/-/-/-	-/-/-/-	+/+/+/+	+/+/+/+	-/-/-/-
**11.**	PF,PO	96,460.8: 160,000	+/+/+/+	-/-/-/-	-/-/-/-	+/+/+/+	+/+/+/+	-/-/-/-	-/-/-/-	+/+/+/+
**12.**	PV,PO	120,576: 32,000	-/-/-/-	+/+/+/+	-/-/-/-	+/+/+/+	-/-/-/-	+/+/+/+	-/-/-/-	+/+/+/+

PF: *P*. *falciparum*, PV: *P*. *vivax*, PM: *P*. *malariae*, PO: *P*. *ovale*, +: positive result,—: negative result

*The samples in which the minor species was identified by ddPCR, but not by real-time PCR

### Diagnostic sensitivity and specificity of ddPCR assay for *Plasmodium* genus and species detection

To validate the diagnostic sensitivity and specificity of ddPCR assays for *Plasmodium* genus and species detection, we used 102 DNA samples obtained from malaria patients which were confirmed as truly positive of *Plasmodium* infection by reference standards. Each reference sample (definitely positive or negative) was confirmed by the three methods: microscopic examination, uPCR, and real-time PCR. We included microscopy as an additional diagnostic to avoid the small chance of missing false negative results when using PCR methods. Results obtained from ddPCR assays of *Plasmodium* genus and species were fully concordant with the binary results (positive or negative) from microscopic examination, uPCR and real-time PCR. The specificity of ddPCR assays were validated using 100 samples obtained from Thai volunteers who had no background of malaria infection, and who were not considered to have been exposed to malaria. These samples were also confirmed to be negative for *Plasmodium* by microscopic examination, uPCR and real-time PCR. The ddPCR assays were negative for all samples. Thus, the ddPCR assays had full concordance for *P*. *falciparum*, *P*. *vivax*, *P*. *malariae* and *P*. *ovale* detection when compared with truly positive and negative results as confirmed by the above 3 methods (Table 2).

**Table 2:** Diagnosis sensitivity and specificity of ddPCR assay for *Plasmodium* genus and species detection.

Results	No. of reference samples	
***Plasmodium* genus detection**		
	Truly positive (Microscopic and uPCR positive)	Truly negative (Microscopic and uPCR negative)
*Plasmodium genus* ddPCR positive	102 (TP)	0 (FP)
*Plasmodium genus* ddPCR negative	0 (FN)	100 (TN)
***P*. *falciparum* detection**		
	Truly positive (Microscopic and real-time PCR positive)	Truly negative (Microscopic and real-time PCR negative)
ddPCR of *P*. *falciparum* positive	44 (TP)	0 (FP)
ddPCR of *P*. *falciparum* negative	0 (FN)	100 (TN)
***P*. *vivax* detection**		
	Truly positive (Microscopic and real-time PCR positive)	Truly negative (Microscopic and real-time PCR negative)
ddPCR of *P*. *vivax* positive	41 (TP)	0 (FP)
ddPCR of *P*. *vivax* negative	0 (FN)	100 (TN)
***P*. *malariae* detection**		
	Truly positive (Microscopic and real-time PCR positive)	Truly negative (Microscopic and real-time PCR negative)
ddPCR of *P*. *malariae* positive	9 (TP)	0 (FP)
ddPCR of *P*. *malariae* negative	0 (FN)	100 (TN)
***P*. *ovale* detection**		
	Truly positive (Microscopic and real-time PCR positive)	Truly negative (Microscopic and real-time PCR negative)
ddPCR of *P*. *ovale* positive	8 (TP)	0 (FP)
ddPCR of *P*. *ovale* negative	0 (FN)	100 (TN)

TP: True positive, FP: False positive, FN: False negative, TN: True negative

### Use of duplex ddPCR assays for subpatent malaria detection

To assess the capability of *Plasmodium* genus and species detection in samples with very low parasitaemia, 32 samples from asymptomatic parasite carriers, which were positive for *Plasmodium* genus results by uPCR [33] in which the specific species could not be determined by real-time PCR assay [25, 41, 42], were assayed by ddPCR for species determination. The ddPCR assay for detection of the *Plasmodium* genus was fully concordant with the uPCR assay. Moreover, the duplex ddPCR for specific *Plasmodium* species detection was able to identify 32 samples at the *Plasmodium* species level: 12 *P*. *vivax* samples (37.5%), 16 *P*. *malariae* samples (50%), 3 mix infection of *P*. *vivax* and *P*. *malariae* (9.375%) and 1 mix infection of *P*. *malariae* and *P*. *ovale* (3.125%).

### Cost and throughput time

Total costs of consumables per one reaction was $5.30 assuming a minimum of 8 reactions. Performing ddPCR requires several steps each with different duration required: reaction set up takes about 30 minutes; droplet generation using Bio-Rad QX100TM Droplet generator and transfer to PCR plate takes about 7 min per 8 samples; conventional PCR takes about 2 hours; droplet reader takes 15 min per eight reactions. Overall, the estimated time was about 7 hours per 96 reactions. In comparison, cost per reaction of the real-time PCR assay is about $3 and the estimated time is about 3 hours per 72 reaction. In case of quantification, a standard curve is required for each round which increases the costs of real-time PCR.

## Discussion

This study aimed to develop a new sensitive assay for detecting and quantifying of *Plasmodium* parasites at the genus and species level using droplet digital PCR. This is the first report describing *Plasmodium* genus quantification using ddPCR. Detection at the genus level could be helpful in determination of parasitaemias in asymptomatic and low density infection. An important application could be for instance better description of the epidemiology of *P*. *malariae*. This parasite can be present in very low parasitaemia for prolonged periods of time, and are often detected as a minor species together with *P*. *falciparum* and *P*. *vivax*. Better ability to detect low parasitaemia of *P*. *malaria*, especially in co-infections, can shed important new light on the endemicity of this parasite.

Using a high volume (1 mL) of sample, the ddPCR assay for *Plasmodium* genus detection had a very low level of detection (11 parasites/mL), even lower than the previously described real-time PCR assays [27], and the uPCR assay (22 parasites/mL) [33]. Another advantage is that the ddPCR assay provides an absolute concentration of DNA targets within each sample without the need for a standard curve for comparison. Prolonged carriage of asymptomatic parasitaemia is thought to contribute importantly to sustaining malaria endemicity, also in low-transmission settings [34]. Its quantitative contribution to malaria transmission is still subject of further study.

Our study follows a recently reported study on a newly developed highly sensitive ddPCR assay for detection and quantification of *P*. *falciparum* and *P*. *vivax* [37]. Our method in addition is able to detect *P*. *malariae* and *P*. *ovale*. Duplex ddPCR for *P*. *falciparum*/ *P*. *vivax* showed higher sensitivity than qPCR to detect *P*. *falciparum*, and equal sensitivity for *P*. *vivax* compared to the previous report [37]. In the current study, the ddPCR assay was developed using duplex reactions for detection and quantification of *P*. *falciparum*/*P*. *vivax* and *P*. *malariae*/*P*. *ovale*. The method was not inferior to a single species singleplex method, and the method was able to detect minority *P*. *malariae* populations in mixed infections, which were in some cases not identified by real-time PCR. The duplex ddPCR assay was also better compared to real-time PCR in detecting very low parasitaemias of all species. The ddPCR assay based on *18S rRNA* gene thus provides a method with improved *Plasmodium* species identification and quantification, with high sensitivity and specificity, which can be used as a research tool to detect subpatent malaria infections with very low parasitaemias.

In addition, the described ddPCR assay provides an accurate method for absolute quantification of all 4 human *Plasmodium* species, not requiring a calibration curve, as also described in the previous study on ddPCR for quantification of *P*. *falciparum* and *P*. *vivax* [37]. In the presence study, quantitative results proved very accurate, when compared to quantification by FACS sorted well-defined parasitaemias.

There were some limitations of this study. Firstly, the duplex ddPCR assay of *Plasmodium* species had lower analytical sensitivity than the ddPCR assay of *Plasmodium* genus. As a result, some samples were identified as *Plasmodium* genus without identification of the specific species. Increased sensitivity of ddPCR could potentially be achieved by using a higher blood volume, but this will require further evaluation. However, in the current format duplex ddPCR assay did have a higher sensitivity to identify the specific *Plasmodium* species compared to real-time PCR. Moreover, compared to real-time PCR, the duplex ddPCR assay had superior performance in identifying minor species populations in mixed infections. Our study suggests that ddPCR can be useful for species identification where real-time PCR is unable to, thus increasing the accuracy of prevalence estimates of specific *Plasmodium* species. Secondly, the sample size where real-time PCR could not identify the specific species whereas ddPCR could, was relatively small (n = 32). Thirdly, we observed an upper limit in the dynamic range for quantification by ddPCR. In samples from clinical cases, with higher parasitaemias, and thus high concentrations of DNA target, quantification will not be possible since all droplets will be positive. The application of ddPCR is thus mainly for detecting low parasitaemias in asymptomatic carriers. For assessment of clinical samples with expected DNA target concentration higher than 9,000–10,000 copies/μl, dilution steps will be necessary.

Finally, the ddPCR assay is expensive and is time consuming but the assay provides a highly sensitive quantitative method not requiring the generation of a standard curve. As a research tool, the duplex ddPCR assay could be cost and time saving compared to singleplex ddPCR assay.

In conclusion, this study describes a new application for the ddPCR assay, to quantify low parasitaemias in subpatent infections as well as for *Plasmodium* species determination in low parasitaemias. The ddPCR assay targeting *18S rRNA* for *Plasmodium* genus detection and quantification could be used for low parasitaemia detection providing absolute quantification of *18S rRNA* gene. The duplex ddPCR assays of *P*. *falciparum*/ *P*. *vivax* and *P*. *malariae*/ *P*. *ovale* could be used for *Plasmodium* species identification in low parasitaemia or mixed infection samples. The assay could be useful as a research tool when absolute quantification of low concentrations *18S rRNA* is required, and mixed infections need identification.

## Supporting information

S1 Fig Two-dimensional (2D) ddPCR results of the duplex ddPCR assay. The duplex ddPCR results in panels a and b were performed using optimized annealing temperatures of 60°C for *P*. *falciparum* and *P*. *vivax* and 52°C for *P*. *malariae* and *P*. *ovale*. The horizontal and vertical lines indicate the fluorescence amplitude cut-offs defining positivity for *P*. *falciparum/P*. *vivax* (panel a) and *P*. *malariae/ P*. *ovale* (panel b).
(PDF)

S1 Table Primers and probes used in *Plasmodium* genus and species detection and quantification. (PDF)

S2 Table Absolute quantification of *18S rRNA* of genus *Plasmodium* (copies/mL). (PDF)

S3 Table Standards for Reporting Diagnostic accuracy studies (STARD) checklist used in this study. (PDF)

S4 Table Identification results of mixed infection samples by duplex ddPCR assay (copies/μl) and real-time PCR assay (Ct-value) (performed in quadruplicate). (PDF)

S5 Table Comparison of ddPCR and real-time PCR assay. (PDF)

S6 Table Absolute quantification of *18S rRNA* of genus *Plasmodium* (copies/mL) using high blood volume (1 mL) and small blood volume (200 μl) for ddPCR assay. (PDF)

S1 File Data on sensitivity and specificity. (XLSX)

S2 File Data on subclinical infections. (XLSX)

S3 File Data on Probit analysis. (XLSX)
