# Supplementary material for: Four human Plasmodium species quantification using droplet digital PCR
Source: PLoS One. 2017 Apr 19;12(4):e0175771. doi: 10.1371/journal.pone.0175771 (PMC5396971; doi:10.1371/journal.pone.0175771)
Supplement: S1 Fig — The duplex ddPCR results in panels a and b were performed using optimized annealing temperatures of 60°C for P. falciparum and P. vivax and 52°C for P. malariae and P. ovale. The horizontal and vertical lines indicate the fluorescence amplitude cut-offs defining positivity for P. falciparum/P. vivax (panel a) and P. malariae/ P. ovale (panel b). (PDF) [file pone.0175771.s001.pdf]

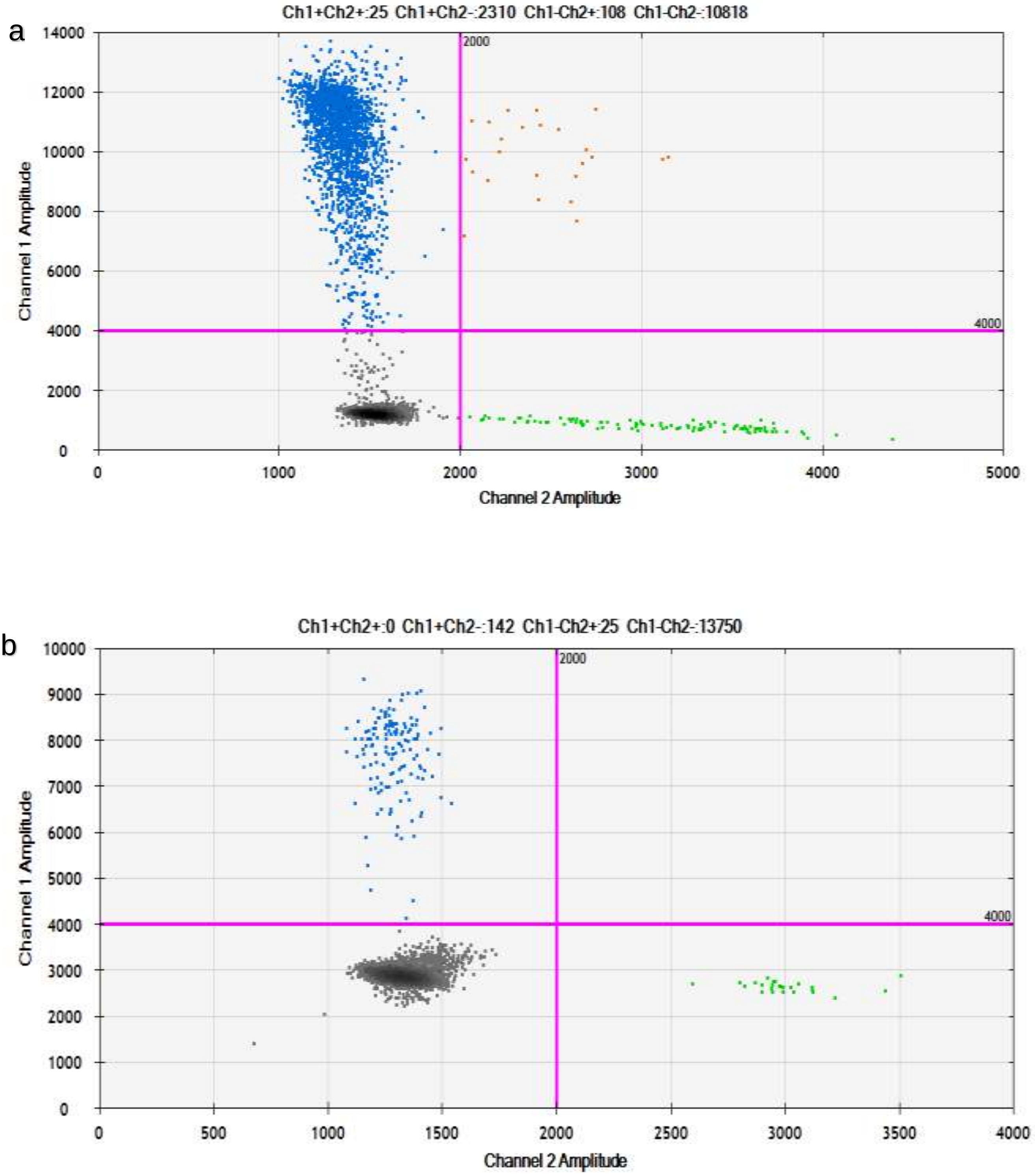

S1 Fig. Two-dimensional (2D) ddPCR results of the duplex ddPCR assay. The duplex ddPCR results in panels a and b were performed using optimized annealing temperatures of 60ºC for *P. falciparum* and *P. vivax* and 52ºC for *P. malariae* and *P. ovale*. The horizontal and vertical lines indicate the fluorescence amplitude cut-offs defining positivity for *P. falciparum*/*P. vivax* (panel a) and *P. malariae*/ *P. ovale* (panel b).
