# Supplementary material for: Four human Plasmodium species quantification using droplet digital PCR
Source: PLoS One. 2017 Apr 19;12(4):e0175771. doi: 10.1371/journal.pone.0175771 (PMC5396971; doi:10.1371/journal.pone.0175771)
Supplement: S1 Table — (PDF) [file pone.0175771.s002.pdf]

S1 Table. Primers and probes used in *Plasmodium* genus and species detection and quantification

Target *18S rRNA* of	Primer/ Sequence(5'-3')	Probe/ Sequence(5'-3')	References
P.falciparum	FAL-F/5'CTTTTGAGAGGTTTTGTTACTTTGAGTAA3'<br>FAL-R/5'TATTCCATGCTGTAGTATTCAAACACAA3'	FAM/<br>5'TGTTCATAACAGACGGGTAGTCATGTTTGAGTTCA3'	(25)
P. vivax	VIV-F/5'ACGCTTCTAGATTAATCCACATAACT3'<br>VIV-R/5'ATTTACTCAAAGTAACAAGGACTTCCAAGC3'	HEX/<br>5'TTCGTATCGACTTTGTGCGCATTTTGC3'	(25)
P. malariae	PLASMO1/5'GTTAAGGGAGTGAAGACGATCAGA3'<br>PLASMO2/5'TTATGAGAAATCAAAGTCTTTGGGTT3'	FAM/<br>5'ATGAGTGTTTCTTTTAGATAG3'	(41)
P.ovale	OVALE-F/5'CCRACTAGGTTTTGGATGAAAVRTTTTT3'<br>PLASMODIUM-R /5'AACCCAAAGACTTTGATTTCTCATAA3'	VIC/<br>5'CRAAAGGAATTYTCTTATT3'	(42)
Genus *Plasmodium*	Forward 5'GCTCTTTCTTGATTTCTTGGATG3'<br>Reverse 5'AGCAGGTTAAGATCTCGTTCG3'	FAM/<br>5'ATGGCCGTTTTTAGTTCGTG3'	(27)

*R=A or G; Y=C or T; V=A or C or
