# Supplementary material for: Four human Plasmodium species quantification using droplet digital PCR
Source: PLoS One. 2017 Apr 19;12(4):e0175771. doi: 10.1371/journal.pone.0175771 (PMC5396971; doi:10.1371/journal.pone.0175771)
Supplement: S2 Table — (PDF) [file pone.0175771.s003.pdf]

S2 Table. Absolute quantification of *18S rRNA* of genus *Plasmodium* (copies/mL)

Number of parasites FACS in blood samples (parasite/mL)	Absolute count from ddPCR (copies/ul) Mean (%CV)	Calculated *18S rRNA* concentration (*18S rRNA* copies /mL of blood) Mean(%CV)	Average ratio of *18S rRNA* (copies/mL) and parasites FACS (parasites/mL) Mean± 2SE
2,000	20.1 (2.96)	4,020 (2.96)	2.01±0.0297
400	3.675(7.16)	735 (7.16)	1.84±0.0657
80	0.655(8.77)	131 (8.77)	1.64±0.0718
16	0.1525 (45.86)	30.5 (45.86)	1.91±0.4371
