# Supplementary material for: Four human Plasmodium species quantification using droplet digital PCR
Source: PLoS One. 2017 Apr 19;12(4):e0175771. doi: 10.1371/journal.pone.0175771 (PMC5396971; doi:10.1371/journal.pone.0175771)
Supplement: S3 Table — (PDF) [file pone.0175771.s004.pdf]

S3 Table. Standards for Reporting Diagnostic accuracy studies (STARD) checklist used in this study

The checklist obtained from http://www.equator-network.org/reporting-guidelines/stard (43).

Section & Topic	No	Item	Reported on page #
**TITLE OR ABSTRACT**			
	1	Identification as a study of diagnostic accuracy using at least one measure of accuracy<br>(such as sensitivity, specificity, predictive values, or AUC)	Page 2
**ABSTRACT**			
	**2**	Structured summary of study design, methods, results, and conclusions<br>(for specific guidance, see STARD for Abstracts)	Page 2
**INTRODUCTION**			
	**3**	Scientific and clinical background, including the intended use and clinical role of the index test	Page 3
	**4**	Study objectives and hypotheses	Page 4
**METHODS**			
Study design	**5**	Whether data collection was planned before the index test and reference standard were performed (prospective study) or after (retrospective study)	Page 4-5
Participants	**6**	Eligibility criteria	Page 4-5
	**7**	On what basis potentially eligible participants were identified<br>(such as symptoms, results from previous tests, inclusion in registry)	Page 4-5
	**8**	Where and when potentially eligible participants were identified (setting, location and dates)	Page 4-5
	**9**	Whether participants formed a consecutive, random or convenience series	Page 4-5
Test methods	**10a**	Index test, in sufficient detail to allow replication	Page 6-8
	**10b**	Reference standard, in sufficient detail to allow replication	Page 9
	**11**	Rationale for choosing the reference standard (if alternatives exist)	Page 9
	**12a**	Definition of and rationale for test positivity cut-offs or result categories<br>of the index test, distinguishing pre-specified from exploratory	Page 6
	**12b**	Definition of and rationale for test positivity cut-offs or result categories<br>of the reference standard, distinguishing pre-specified from exploratory	Page 9
	**13a**	Whether clinical information and reference standard results were available<br>to the performers/readers of the index test	Reference standard results were compared with ddPCR results in Table 2.
	**13b**	Whether clinical information and index test results were available<br>to the assessors of the reference standard	Index test results were compared with reference standard results in Table 2.
Analysis	**14**	Methods for estimating or comparing measures of diagnostic accuracy	Page 9-11
	**15**	How indeterminate index test or reference standard results were handled	There is no indeterminate index test or reference standard results.
	**16**	How missing data on the index test and reference standard were handled	There is no missing data.
	**17**	Any analyses of variability in diagnostic accuracy, distinguishing pre-specified from exploratory	Page 13
	**18**	Intended sample size and how it was determined	Page 4
**RESULTS**			
Participants	**19**	Flow of participants, using a diagram	Figure 1
	**20**	Baseline demographic and clinical characteristics of participants	Page 4
	**21a**	Distribution of severity of disease in those with the target condition	No
	**21b**	Distribution of alternative diagnoses in those without the target condition	No
	**22**	Time interval and any clinical interventions between index test and reference standard	Page 14<br>There is no clinical intervention.
Test results	**23**	Cross tabulation of the index test results (or their distribution)<br>by the results of the reference standard	Page 13<br>Table 2
	**24**	Estimates of diagnostic accuracy and their precision (such as 95% confidence intervals)	Page 9-10
	**25**	Any adverse events from performing the index test or the reference standard	No
**DISCUSSION**			

	**26**	Study limitations, including sources of potential bias, statistical uncertainty, and generalizability	Page 16
	**27**	Implications for practice, including the intended use and clinical role of the index test	Page 17
**OTHER INFORMATION**			
	**28**	Registration number and name of registry	No
	**29**	Where the full study protocol can be accessed	No
	**30**	Sources of funding and other support; role of funders	Page 17
