# Supplementary material for: Four human Plasmodium species quantification using droplet digital PCR
Source: PLoS One. 2017 Apr 19;12(4):e0175771. doi: 10.1371/journal.pone.0175771 (PMC5396971; doi:10.1371/journal.pone.0175771)
Supplement: S4 Table — (PDF) [file pone.0175771.s005.pdf]

S4 Table. Identification results of mixed infection samples by duplex ddPCR assay (copies/μl) and real-time PCR assay (Ct-value) (performed in quadruplicate)

Sample No.	Artificial Mixed of	Estimated parasites concentration (Parasites/mL)	Real time PCR results mean Ct (%CV)				Duplex ddPCR results mean copies/ul of ddPCR reaction (%CV)			
			P.falciparum	*P.vivax*	*P.malariae*	*P.ovale*	*P.falciparum*	*P.vivax*	*P.malariae*	*P.ovale*
**1.**	PF,PV	96,460.8 : 1205.76	27.47 (1.36)	34.20 (0.75)	negative	negative	39.15 (8.97)	0.48 (37.36)	negative	negative
**2.**	PF,PV	19,292.16 : 1205.76	30.32 (1.21)	33.79 (1.10)	negative	negative	6.54 (15.592)	0.68 (29.12)	negative	negative
**3.**	PF,PV	3,858.43 : 1,205.75	32.39 (2.84)	33.93 (1.59)	negative	negative	1.25 (16.65)	0.67 (18.96)	negative	negative
**4.**	PF,PV	4,823.04 : 120,576	34.14 (2.21)	27.75 (1.63)	negative	negative	0.92 (35.10)	54.93 (4.91)	negative	negative
**5.**	PF,PV	4,823.04 : 24,115.2	33.65 (2.04)	30.29 (1.52)	negative	negative	0.81 (25.10)	12.31 (15.59)	negative	negative
**6.**	PF,PV	4,823.04 :4,823.04	33.86 (1.17)	33.24 (1.55)	negative	negative	0.87 (13.82)	2.74 (11.68)	negative	negative
**7.***	PF,PM	482,304:128,000	24.76 (0.92)	negative	negative	negative	213.38 (2.85)	negative	8.58 (6.41)	negative
**8.***	PF,PM	96,460.8:128,000	27.50 (1.06)	negative	negative	negative	40.60 (4.6)	negative	8.23 (11.80)	negative
**9.***	PV,PM	120,576:128,000	negative	27.77 (1.18)	negative	negative	negative	56.90 (1.7)	8.68 (10.99)	negative
**10.**	PV,PM	4,823.04:128,000	negative	32.93 (1.96)	31.17(0.95)	negative	negative	2.87 (7.16)	8.58 (7.76)	negative
**11.**	PF,PO	96,460.8 :160,000	27.72 (1.47)	negative	negative	32.48(1.45)	40.74 (5.62)	negative	negative	3.58 (8.35)
**12.**	PV,PO	120,576 :32,000	negative	32.84 (0.9)	negative	34.92(0.75)	negative	2.92 (7.07)	negative	0.69 (13.87)

PF: *P. falciparum*, PV: *P. vivax*, PM: *P.malariae*, PO: *P.ovale*, %CV: Coefficient of Variation

*The samples in which the minor species was identified by ddPCR, but not by real-time PCR

S4 Table. (Cont.) Identification results of mixed infection samples by duplex ddPCR assay (copies/μl) and real-time PCR assay (Ct-value) (performed in quadruplicate)

Sample No.	Artificial Mixed of	Estimated parasites concentration (Parasites/mL)	Results from Real-time PCR assay (Ct) performed in quadruplicate															
			for *P.falciparum* detection				for *P.vivax* detection				for *P.malariae* detection				for *P.ovale* detection			
			1st	2nd	3rd	4th	1st	2nd	3rd	4th	1st	2nd	3rd	4th	1st	2nd	3rd	4th
1	PF,PV	96,460.8 : 1205.76	27.46	27.27	27.14	27.99	34.31	34.51	33.92	34.09	neg	neg	neg	neg	neg	neg	neg	neg
2	PF,PV	19,292.16 : 1205.76	30.7	30.16	29.89	30.54	33.61	33.48	33.75	34.32	neg	neg	neg	neg	neg	neg	neg	neg
3	PF,PV	3,858.43 : 1,205.75	32.66	33.31	32.45	31.12	34.48	34.27	33.32	33.65	neg	neg	neg	neg	neg	neg	neg	neg
4	PF,PV	4,823.04 : 120,576	34.91	33.92	34.55	33.19	28.12	27.4	28.16	27.32	neg	neg	neg	neg	neg	neg	neg	neg
5	PF,PV	4,823.04 : 24,115.2	33.15	33.83	33.08	34.53	30.02	30.35	30.91	29.87	neg	neg	neg	neg	neg	neg	neg	neg
6	PF,PV	4,823.04 :4,823.04	33.39	33.68	34.24	34.13	32.69	33.87	33.41	32.98	neg	neg	neg	neg	neg	neg	neg	neg
7.	PF,PM	482,304:128,000	24.86	24.48	24.68	25.01	neg	neg	neg	neg	neg	neg	neg	neg	neg	neg	neg	neg
8.	PF,PM	96,460.8:128,000	27.56	27.27	27.29	27.89	neg	neg	neg	neg	neg	neg	neg	neg	neg	neg	neg	neg
9.	PV,PM	120,576:128,000	neg	neg	neg	neg	28.08	27.52	28.02	27.45	neg	neg	neg	neg	neg	neg	neg	neg
10	PV,PM	4,823.04:128,000	neg	neg	neg	neg	32.29	33.48	33.49	32.46	31.59	30.95	31.15	30.98	neg	neg	neg	neg
11	PF,PO	96,460.8 :160,000	27.96	27.82	27.12	27.98	neg	neg	neg	neg	neg	neg	neg	neg	32.21	33.05	32.65	31.99
12	PV,PO	120,576 :32,000	neg	neg	neg	neg	32.97	32.52	32.69	33.19	neg	neg	neg	neg	34.62	35.25	34.85	34.98

Sample No.	Artificial Mixed of	Estimated parasites concentration (Parasites/mL)	Results from ddPCR assay (copies/ul of ddPCR) performed in quadruplicate															
			for *P.falciparum* detection				for *P.vivax* detection				for *P.malariae* detection				for *P.ovale* detection			
			1st	2nd	3rd	4th	1st	2nd	3rd	4th	1st	2nd	3rd	4th	1st	2nd	3rd	4th
1	PF,PV	96,460.8 : 1205.76	42.2	38.4	41.5	34.5	0.34	0.67	0.31	0.58	neg	neg	neg	neg	neg	neg	neg	neg
2	PF,PV	19,292.16 : 1205.76	7.8	5.4	6.8	6.14	0.87	0.83	0.54	0.48	neg	neg	neg	neg	neg	neg	neg	neg
3	PF,PV	3,858.43 : 1,205.75	1.3	1	1.5	1.2	0.53	0.59	0.74	0.8	neg	neg	neg	neg	neg	neg	neg	neg
4	PF,PV	4,823.04 : 120,576	0.82	0.74	1.4	0.72	56.3	55.4	51	57	neg	neg	neg	neg	neg	neg	neg	neg
5	PF,PV	4,823.04 : 24,115.2	1.05	0.85	0.77	0.56	11.8	10.45	15	12	neg	neg	neg	neg	neg	neg	neg	neg
6	PF,PV	4,823.04 :4,823.04	0.99	0.94	0.83	0.72	2.9	2.4	2.55	3.1	neg	neg	neg	neg	neg	neg	neg	neg
7.	PF,PM	482,304:128,000	217	206	219.5	211	neg	neg	neg	neg	16.4	18.6	17.4	16.2	neg	neg	neg	neg
8.	PF,PM	96,460.8:128,000	42.2	38.4	39.7	42.1	neg	neg	neg	neg	17	18.8	15.8	14.2	neg	neg	neg	neg
9.	PV,PM	120,576:128,000	neg	neg	neg	neg	56.3	57.4	55.9	58	17	18.6	14.8	19	neg	neg	neg	neg
10	PV,PM	4,823.04:128,000	neg	neg	neg	neg	2.9	3.1	2.6	2.9	16.4	18.8	17.6	15.8	neg	neg	neg	neg
11	PF,PO	96,460.8 :160,000	42.2	39.75	38	43	neg	neg	neg	neg	neg	neg	neg	neg	7.8	6.4	7.4	7
12	PV,PO	120,576 :32,000	neg	neg	neg	neg	2.9	3.2	2.7	2.9	neg	neg	neg	neg	1.6	1.34	1.14	1.42

PF: *P. falciparum*, PV: *P. vivax*, PM: *P.malariae*, PO: *P.ovale*, neg: negative result
