# Supplementary material for: Four human Plasmodium species quantification using droplet digital PCR
Source: PLoS One. 2017 Apr 19;12(4):e0175771. doi: 10.1371/journal.pone.0175771 (PMC5396971; doi:10.1371/journal.pone.0175771)
Supplement: S5 Table — (PDF) [file pone.0175771.s006.pdf]

S5 Table. Comparison of ddPCR and real-time PCR assay.

Topic	ddPCR assay	Real-time PCR and uPCR (33)
Principle	ddPCR assay is a partial PCR based on water-oil emulsion droplet technology that all components in a reaction are divided into 20,000 droplets. The DNA target concentration is calculated from the number of positive and negative droplets using Poisson statistics.<br>(on page 2-3)	Real-time PCR is a PCR based method used to monitor the progress of a PCR reaction in real time. The amount of DNA target is estimated via "sigmoidal" or "logistic" curve fitting models.
Quantitative	Yes, the number of positive and negative droplets in a reaction were analyzed using Poisson statistics.<br>(on page 2-3)	Yes, quantity of DNA target is directly proportional to the exponential phase of PCR.
Advantages – disadvantages	- Not require references or standards for quantification assay (1,2)<br>(on page 2-3)<br><br>- Analytical sensitivity to detect *Plasmodium* genus is 11 parasites/mL analyzed by Probit analysis<br>(obtained from this study)<br>(on page 10 line 228)<br><br>- Highly precision to quantitate DNA targets<br>(obtained from this study)<br>(Page 9-10 line 232-237 and in S2 Table)<br><br>- Higher tolerant to inhibitors (1,2)<br><br>- Higher ability to analyze the complex mixtures. This study showed the ability of ddPCR to identify minor parasites in mixed samples.<br>(obtained from this study)<br>(Page 11 line 269-276)<br><br>- Cost $5.30 per sample<br>(obtained from this study)<br>(Page 14 line 311)<br><br>- Takes about 7 hours per 96 reactions (Time consuming)<br>(obtained from this study)<br>(Page 14 line 312-316)<br><br>- Post ddPCR product unable to perform DNA sequencing analysis	- Require references or standards<br><br>- Analytical sensitivity 22 parasites/mL analyzed by Probit analysis<br>(experiment was run in parallel in this study and previous study (33))<br>(Figure3)<br><br>Precision to quantitate DNA targets<br><br>- Tolerant to inhibitors<br><br>- Lower ability to analyze the complex mixtures. This study showed that real-time PCR assay was unable to identify minor parasites in mixed samples.<br><br>- Cost $3.0 per sample<br>(Page 14 line 317)<br><br>- Time consuming about 3 hours per 72 reactions<br>(Page 14 line 316-317)<br><br>- Post PCR product able to perform DNA sequencing analysis
