# Supplementary material for: Four human Plasmodium species quantification using droplet digital PCR
Source: PLoS One. 2017 Apr 19;12(4):e0175771. doi: 10.1371/journal.pone.0175771 (PMC5396971; doi:10.1371/journal.pone.0175771)
Supplement: S6 Table — (PDF) [file pone.0175771.s007.pdf]

S6 Table. Absolute quantification of *18S rRNA* of genus *Plasmodium* (copies/mL) using high blood volume (1 mL) and small blood volume (200 µl) for ddPCR assay.

FACS Samples concentration (parasite/mL)	High blood volume<br>1mL of blood extracted and then concentrated to 40 µl of DNA samples<br>(4 µL to ddPCR assay)			Small blood volume<br>200 µl of whole blood extracted to 200 µl of DNA samples<br>(4 µL to ddPCR assay)		
	18S rRNA concentration from ddPCR assay (copies/µl) Mean (%CV) (n=16)	*18S rRNA* concentration (copies/mL) Mean	Ratio of *18S rRNA* (copies per mL) and parasites FACS (parasites/mL)	*18S rRNA* concentration from ddPCR assay (copies/µl) Mean (%CV) (n=4)	*18S rRNA* concentration (copies/mL) Mean	Ratio of *18S rRNA* (copies per mL) and parasites FACS (parasites/mL)
2,000	20.1 (2.96)	4,020	2.01	0.8675 (5.75)	4,338	2.17
400	3.675 (7.16)	735	1.84	0.15 (27.22)	750	1.88
80	0.655 (8.77)	131	1.64	negative	negative	negative
16	0.1525 (45.86)	30.5	1.91	negative	negative	negative
